# Integrative metabolomic and transcriptomic analysis reveals difference in glucose and lipid metabolism in the longissimus muscle of Luchuan and Duroc pigs

**DOI:** 10.3389/fgene.2023.1128033

**Published:** 2023-04-06

**Authors:** Liyan Deng, Wangchang Li, Weiwei Liu, Yanwen Liu, Bingkun Xie, Martien A. M. Groenen, Ole Madsen, Xiaogan Yang, Zhonglin Tang

**Affiliations:** ^1^ Kunpeng Institute of Modern Agriculture at Foshan, Foshan, China; ^2^ Guangdong Laboratory for Lingnan Modern Agriculture, Shenzhen Branch, Agricultural Genomics Institute at Shenzhen, Chinese Academy of Agricultural Sciences, Shenzhen, China; ^3^ The Key Laboratory of Livestock and Poultry Bioomics of the Ministry of Agriculture and Rural Affairs, Agricultural Genomics Institute at Shenzhen, Chinese Academy of Agricultural Sciences, Shenzhen, China; ^4^ Animal Breeding and Genomics, Wageningen University and Research, Wageningen, Netherlands; ^5^ Guangxi Key Laboratory of Animal Breeding and Disease Control, College of Animal Science and Technology, Guangxi University, Nanning, Guangxi, China; ^6^ GuangXi Engineering Centre for Resource Development of Bama Xiang Pig, Bama, China; ^7^ Guangxi Key Laboratory of Livestock Genetic Improvement, Guangxi Institute of Animal Sciences, Nanning, China

**Keywords:** Duroc, Luchuan, metabolic profiling, RNA-seq, lipid metabolism, glycolysis, AMPK signaling pathway, fructose and mannose metabolism

## Abstract

Luchuan pig, an obese indigenous Chinese porcine breed, has a desirable meat quality and reproductive capacity. Duroc, a traditional western breed, shows a faster growth rate, high feed efficiency and high lean meat rate. Given the unique features these two porcine breeds have, it is of interest to investigate the underlying molecular mechanisms behind their distinctive nature. In this study, the metabolic and transcriptomic profiles of longissimus dorsi muscle from Duroc and Luchuan pigs were compared. A total of 609 metabolites were identified, 77 of which were significantly decreased in Luchuan compared to Duroc, and 71 of which were significantly elevated. Most differentially accumulated metabolites (DAMs) upregulated in Luchuan were glycerophospholipids, fatty acids, oxidized lipids, alcohols, and amines, while metabolites downregulated in Luchuan were mostly amino acids, organic acids and nucleic acids, bile acids and hormones. From our RNA-sequencing (RNA-seq) data we identified a total of 3638 differentially expressed genes (DEGs), 1802 upregulated and 1836 downregulated in Luchuan skeletal muscle compared to Duroc. Combined multivariate and pathway enrichment analyses of metabolome and transcriptome results revealed that many of the DEGs and DAMs are associated with critical energy metabolic pathways, especially those related to glucose and lipid metabolism. We examined the expression of important DEGs in two pathways, AMP-activated protein kinase (AMPK) signaling pathway and fructose and mannose metabolism, using Real-Time Quantitative Reverse Transcription PCR (qRT-PCR). Genes related to glucose uptake, glycolysis, glycogen synthesis, fatty acid synthesis (*PFKFB1, PFKFB4, MPI, TPI1, GYS1, SLC2A4, FASN, IRS1, ULK1*) are more activated in Luchuan, while genes related to fatty acid oxidation, cholesterol synthesis (*CPT1A, HMGCR, FOXO3*) are more suppressed. Energy utilization can be a decisive factor to the distinctive metabolic, physiological and nutritional characteristics in skeletal muscle of the two breeds we studied. Our research may facilitate future porcine breeding projects and can be used to reveal the potential molecular basis of differences in complex traits between various breeds.

## Introduction

China is the largest pork consuming and producing country in the world and has a great variety of native porcine breeds. On one hand, Luchuan pig, as one of the most famous indigenous Chinese porcine breeds, is an obese breed that has a desirable meat quality and reproductive capacity. On the other hand, Duroc, a traditional breed developed from the United States, shows a faster growth rate, high feed efficiency, strong reproductive characteristics and relatively high lean meat rate. However, it has been reported that pork from Duroc, Landrace and Yorkshire crosses has the tendency to be of lower quality (Faucitano et al., 2010). Research has shown that meat quality is related to muscle fiber characteristics and intramuscular fat content (IMF) (Joo et al., 2013; Zhang et al., 2021). Larger muscle fibers can form larger muscle bundles (Chen et al., 2007; Kokoszynski et al., 2019), and therefore affect the tenderness of meat. The IMF content, normally referred as the lipid droplets accumulated in muscle fibers and fat cells, is essential for meat tenderness and meat flavor (Katsumata, 2011; Hausman et al., 2014; Sun, Chen et al., 2018). Given the respective advantages and unique features that the Luchuan and Duroc pigs have, it is of interest to investigate the underlying molecular mechanisms behind their utterly distinctive nature.

Metabolites are fundamental for the formation of phenotypes. Understanding the metabolite composition is key to illustrate the mechanisms underlying specific biological traits. Metabolomic profiling is a technique that identifies and quantifies low weight molecules or metabolites in a given biological system. It has frequently been applied in research that tracks changes in metabolites and their associated biochemical pathways in relation to, for example, disease (Hasin et al., 2017). In animal breeding, a previous study utilized metabolomic networks to identify hub metabolites and pathways under different feeding conditions in Duroc and Landrace pigs, which has provided potential biomarkers for improving feed efficiency (Carmelo et al., 2020). It is noticeable that the integration of metabolic profiling and other omics approaches such as transcriptomics can be very powerful to study complex traits and biological problems (Hao et al., 2021).

Metabolic analysis is typically categorized as two complementary methods: Targeted and untargeted. The targeted approach focuses on identifying and quantifying selected metabolites, while the untargeted approach measures all the metabolites of a biological system (Man et al., 2021). A novel, widely targeted metabolomics method was developed that could detect hundreds of targeted metabolites (Sawada et al., 2009). Compared to the total scan ESI (electrospray ionisation) based non-targeted metabolomics (Matsuda et al., 2012), widely targeted metabolomics based on multiple reaction monitoring (MRM) is a very sensitive and accurate method for the measurement of targeted metabolites (Chen et al., 2013). LC-MS/MS (liquid chromatography-tandem mass spectrometry), one of the commonly used techniques for metabolic analysis, combines physical separation capabilities of liquid chromatography with the mass analysis capabilities of mass spectrometry. UPLC-MS/MS (ultraperformance LC-MS/MS), an upgraded method compared to LC-MS/MS, produces significant improvements in sensitivity, speed, and resolution (Churchwell et al., 2005). A recent study adopted a UPLC-MS/MS based metabolomic approach to reveal metabolic profiles of five commercial truffle species, and identified the metabolites and pathways that were different among these species (Li et al., 2019). Another research team compared the longissimus dorsi lipidomes among cattle-yak, yak, and cattle, and detected 296 lipids using the same UPLC-MS/MS approach. They uncovered the variance in energy metabolism and lipid nutrition quality between plateau cattle (cattle-yak and yak) and cattle muscle samples (Gu et al., 2021).

In this research, we incorporated UPLC-MS/MS based widely targeted metabolomics and RNA-sequencing method to compare the two porcine breeds, Duroc and Luchuan. We aimed to investigate metabolic and gene expression differences between the two breeds and identify those impactful pathways involved. Our study provides further insight in the molecular basis underlying the characteristic differences between these representative breeds.

## Materials and methods

### Animals

The pigs in this study were obtained from a commercial pig farm in Yangjiang city, Guangdong province, China. All piglets were fed the same ensilage-concentrate fodder, provided with water *ad libitum*, and were raised under the same environment. The pigs were slaughtered at the age of 300 days, with an average weight of 108.5 kg (Duroc) and 69 kg (Luchuan), respectively. A total of 20 longissimus dorsi muscle samples were collected in the same location from each animal (10 samples per breed), and were immediately frozen in liquid nitrogen. The samples were then stored at −80°C until metabolic extraction. All animal procedures were performed according to protocols approved by the Biological Studies Animal Care and Use Committee in Guangdong Province, China, and guidelines for the Care and Use of Experimental Animals established by the Ministry of Agriculture and Rural Affairs of China.

### Sample preparation and metabolite extraction

The muscle tissue was thawed on ice and 50 ± 2 mg per sample was homogenized with cold beads at 30 Hz for 3 min. The homogenate was added with 1 mL 70% methanol and whirled for 5 min, and subsequently centrifuged at 12,000 rpm at 4°C for 10 min. After centrifugation, 400 μL of supernatant was transferred into a new 1.5 mL Eppendorf tube and then stored at −20°C overnight. The extracts were then centrifuged at 12,000 rpm, at 4°C, for 3 min. We took 400 μL of supernatant from each sample for the LC-MS/MS analysis.

### ESI-Q TRAP-MS/MS analysis

The metabolite extracts were analyzed using an LC-ESI-MS/MS system (UPLC, ExionLC AD system, https://www.sciex.com/, Shanghai, China; MS, SCIEX Triple Quad 6500+ LC-MS/MS system, https://www.sciex.com/, Shanghai, China). Precisely 2 μL of aliquots were injected into a Waters ACQUITY UPLC HSS T3 C18 column (1.8 μm, 2.1 mm*100 mm). The UPLC solvents used were purified water (containing 0.1% formic acid, solvent A) and acetonitrile (containing 0.1% formic acid, solvent B). The column temperature was 40°C and the flow rate was set at 0.4 mL/min. The gradient program was: 95:5 V/V at 0 min, 10:90 V/V at 10.0 min, 10:90 V/V at 11.0 min, 95:5 V/V at 11.1 min, 95:5 V/V at 14.0 min.

LIT and triple quadrupole (QQQ) scans were obtained using a triple quadrupole-linear ion trap mass spectrometer (QTRAP), SCIEX Triple Quad 6500+ LC-MS/MS system, equipped with an ESI Turbo Ion-Spray interface. The experiment was performed in positive and negative ion mode and controlled by Analyst 1.6.3 software (AB Sciex). The ESI source operation parameters were: Source temperature 500°C; ion spray voltage (IS) 5,500 V (positive), −4,500 V (negative); ion source gas I (GSI), gas II (GSII), curtain gas (CUR) were set at 55, 60, and 25.0 psi, respectively; the collision gas (CAD) was set at high. We adopted 10 and 100 μmol/L of polypropylene glycol solutions in QQQ and LIT modes for Instrument tuning and mass calibration. To produce maximal signal, collision energy (CE) and de-clustering potential (DP) were optimized for each precursor–product ion transition (Chen et al., 2013). A specific set of MRM transitions would be monitored for each period based on the metabolites eluted within the period.

### Metabolite identification and statistical analysis

The MS data were processed, and the metabolites were annotated using the Metware in-house MS2 spectral tag (MS2T) library (Wuhan Metware Biotechnology Co., Ltd.; http://www.metware.cn, Wuhan, China). The relative quantitation of metabolites was then performed with unsupervised principal component analysis (PCA) using the *prcomp* function embedded in R (https://www.r-project.org/). The HCA (hierarchical cluster analysis) results of samples and metabolites were plotted and presented as heatmaps with dendrograms, while pearson correlation coefficients (PCC) between samples were calculated by the *cor* function in R and presented as heatmaps. Both HCA and PCC were carried out by *ComplexHeatmap* (https://github.com/jokergoo/ComplexHeatmap) in R. For HCA, normalized signal intensities of metabolites (unit variance scaling) are visualized as a color spectrum.

Subsequently, supervised multiple regression orthogonal partial least-squares discriminant analysis (OPLS-DA) was performed with *ropls* in R (Thevenot et al., 2015). The data was log transformed (log2) and mean-centered prior to OPLS-DA. The models were validated with 200 permutation tests to prevent model overfitting. Significant differentially accumulated metabolites (DAMs) between the two breeds were filtered by the following criteria: Fold change ≥2 would be considered as upregulated and fold change ≤0.5 would be considered as downregulated (Luchuan vs. Duroc); the variable importance in projection (VIP) score of metabolites extracted from OPLS-DA result are greater than 1. The significance of difference of the metabolites between the two breeds was examined by an independent-sample *t*-test (*p* = 0.05). Score plots and permutation plots were generated using the R package MetaboAnalystR (https://www.metaboanalyst.ca/).

Annotated metabolites were mapped to the Kyoto Encyclopedia of Genes and Genomes (KEGG) pathway database (http://www.kegg.jp/kegg/pathway.html). A pathway enrichment analysis of significantly regulated metabolites was performed on Metabolite Sets Enrichment Analysis (MSEA; https://www.msea.ca/). The significance of a pathway was determined by hypergeometric test’s *p*-values.

### RNA-seq and integration analysis

Longissimus dorsi muscle tissues were collected from the animals mentioned in the previous section, and total RNA was extracted from each sample using TRIzol reagent (Invitrogen). The RNA-seq libraries were constructed according to Illumina’s standard operating protocols and pair-end RNA-seq was performed on Illumina NovaSeq 6000, generating data with read length of 150bp. All transcriptome datasets were stored in the China National GenBank (https://db.cngb.org/) Nucleotide Sequence Archive (CNSA) under accession number CNP0001159. We downloaded the pig reference genome sequence and gene annotation files from Ensembl (release 95), and our RNA-seq reads were aligned to the pig reference genome (*Sus scrofa* 11.1) using TopHat v2.1.0 (default settings). The number of mapped genes were counted by Feature Counts v1.6.2 and were normalized to TPM. Differential gene analysis was performed with DEGseq2 (v1.38.1, default settings), and the significantly differentially expressed genes were identified based on the following criteria: |log2(fold change)| ≥ 1 and q < 0.05. Three biological replicates were used for DEGseq2 analysis.

Pearson correlation analysis was performed between DEGs and DAMs using the normalized expression of genes and metabolite concentration. Pearson correlation coefficient (PCC) ≥ 0.8 and *p* < 0.05 (∗) or *p* < 0.01 (∗∗) was used to indicate significance. Gene Ontology (GO) enrichment analysis of DEGs was performed using DAVID v6.8 (http://david.abcc.ncifcrf.gov/) and KOBAS (http://kobas.cbi.pku.edu.cn/kobas3/) was used for KEGG pathway enrichment analysis. We selected genes and metabolites that are significant in our functional enrichment and correlation test, and pathways that are present in both of our metabolomic and transcriptomic analyses. In order to explore our metabolomic and transcriptomic dataset and find out potential DEGs related to breed characteristics, we narrowed down to two metabolic pathways that were significant in the transcriptomic analysis and with the lowest *p*-values in the metabolomic analysis. Heatmap of DEGs and DAMs was plotted to visualize the degree of correlation between genes and metabolites.

### Validation of gene expression

We obtained three duplicate tissue samples from each pig and extracted total RNA using TRIzol reagent (OMEGA, United States; Genstar, Beijing, China). Reverse transcription was completed by a first strand cDNA synthesis kit (Takara, Dalian, China). Thermal cycling conditions were as follows: 95°C for 1 min, followed by 40 cycles of 10 s at 95°C, 34 s at 60°C, and 1 min at 60°C. The primers used in this study were designed by primer 5.0 (Supplementary Table S1). We examined the expression of 13 genes presented in AMPK signaling pathway and Fructose and mannose metabolism, and those genes were significantly associated with the DAMs found in these two pathways. We selected *β*-Actin gene as reference in all our qRT-PCR experiments, and fold change was calculated by means of the formula 2^−ΔΔCT^. Data processing, calculation and histogram drawing was finished by Microsoft Excel 2019, and variance analysis was done with Prism 8.0.2. All data was expressed as mean ± SEM. Statistical analysis was performed by the unpaired two-tailed Student’s *t*-test. *p* < 0.05 (∗) or *p* < 0.01 (∗∗) was used to indicate significance.

## Results

### Widely targeted metabolic profiling of Luchuan and Duroc samples

Widely targeted UPLC-MS/MS approach was applied for comprehensive metabolic profiling of 20 longissimus dorsi muscle samples, from 10 Luchuan pigs and 10 Duroc pigs, respectively. A total of 609 metabolites were detected, including amino acids, lipids, fatty acids and other primary and secondary metabolites that were enriched in skeletal muscle.

### Multivariate analysis of metabolites

**FIGURE 1:**
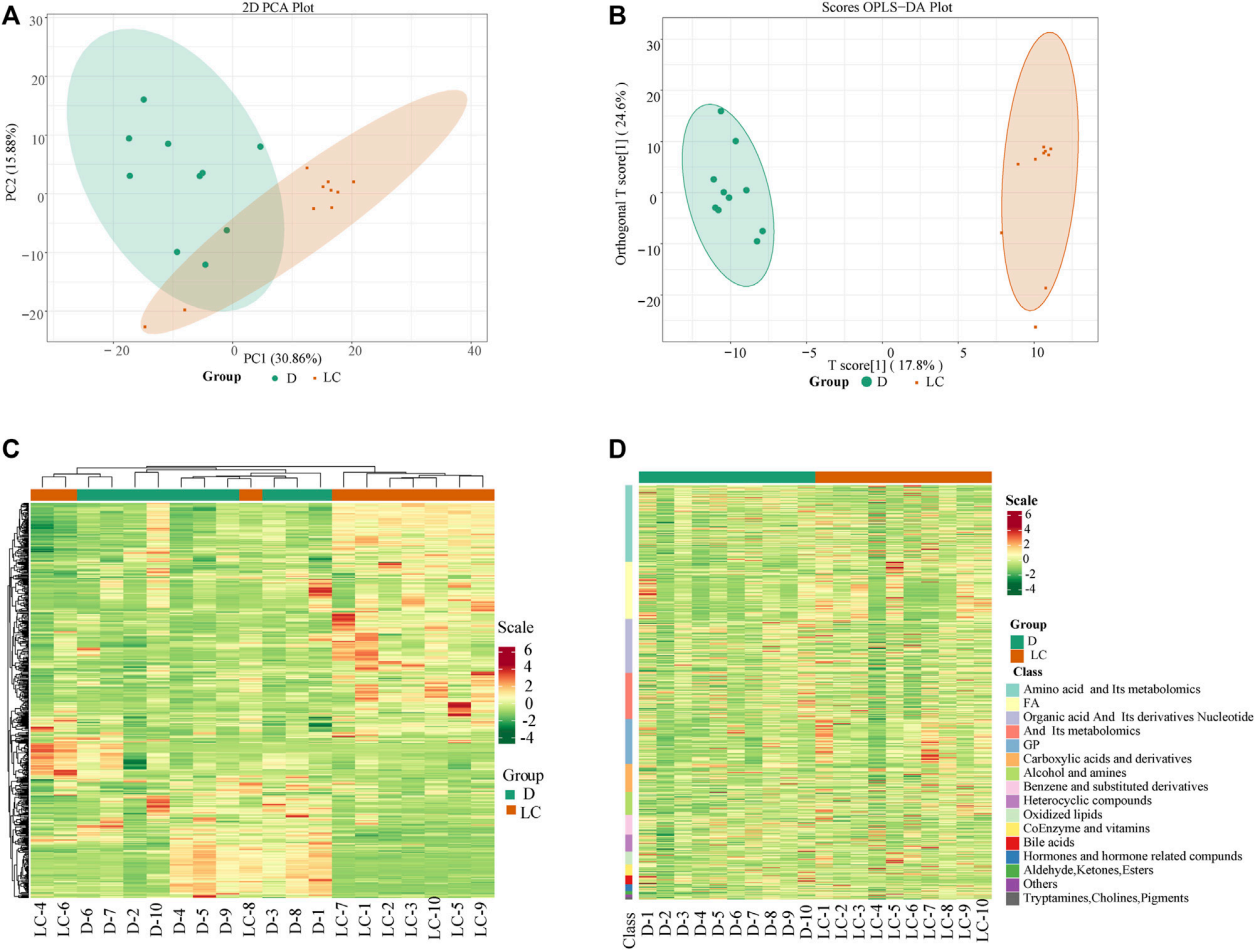
Multivariate and cluster analyses results of Duroc and Luchuan skeletal muscle metabolites. **(A)** PCA analysis of metabolites detected in Duroc and Luchuan samples. Duroc is highlighted in green and Luchuan in orange. **(B)** OPLS-DA score plot demonstrates separation of the Duroc and Luchuan groups. **(C)** Hierarchical cluster analysis of metabolites from Luchuan and Duroc samples. The color represents accumulation of metabolites, from low (green) to high (red). The Z score scale marks the deviation from the mean by standard deviation units. **(D)** Heatmap of all DAMs. The metabolites were classified into 16 classes, and the colors display the abundance of metabolites.

We performed PCA analysis based on the 609 metabolites that were identified, and the two-dimension PCA plot showed separation and difference among most of the samples on both principal components, PC1 and PC2 (Figure 1A). Our OPLS-DA model displayed satisfactory modeling and predictive abilities with 1 predictive component and 2 orthogonal components (*R* ^2^X = 0.629, *R* ^2^Ycum = 0.986, *Q* ^2^cum = 0.888). Split of Luchuan and Duroc was observed on the *x*-axis of the OPLS-DA score plot, suggesting that the two porcine breeds contributed to their different metabolic profile in skeletal muscle (Figure 1B). The respective VIP of metabolites were used to discriminate DAMs, as it reflects the importance of variables in the OPLS-DA model.

Subsequently, a hierarchical cluster analysis was conducted and the result shows that the two breeds (Duroc, green; Luchuan, orange) are in separate clusters (Figure 1C). The DAMs were categorized into 16 classes. The expression of amino acids and their derivatives, organic acids, nucleotide and their derivatives, bile acids, and hormones, is substantially lower in the Luchuan group compared to the Duroc group (Figure 1D). Meanwhile, the concentration of glycerophospholipids, fatty acids, oxidized lipids, alcohols, and amines from the Luchuan group is considerably higher than the Duroc group. Our PCA and cluster analyses results suggest that the two studied porcine breeds have their own distinctive metabolic profile.

### Differential analysis, functional annotation and pathway enrichment of significant metabolites

**FIGURE 2:**
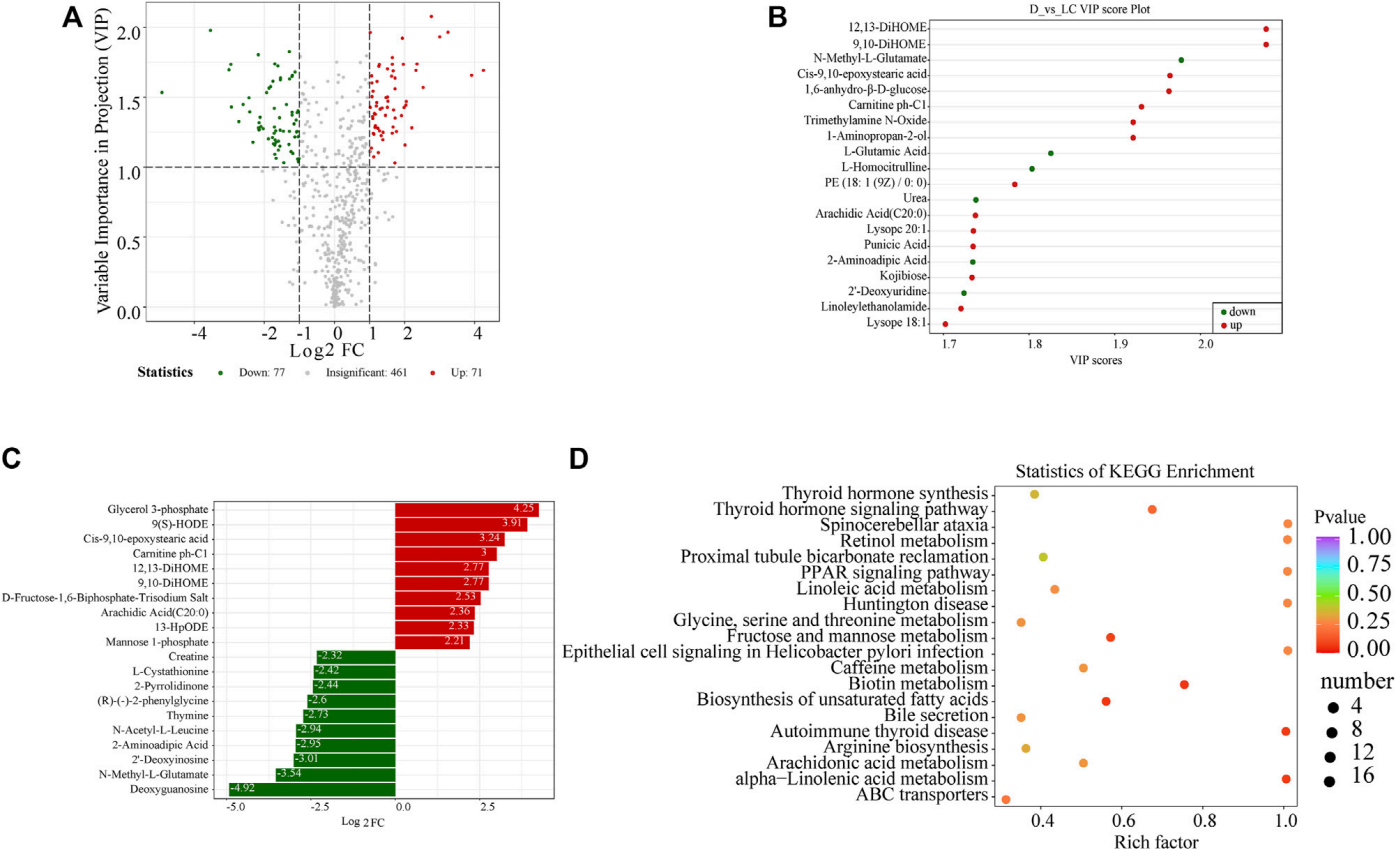
Significant DAMs in Luchuan and Duroc. **(A)** Volcano plot of all 609 metabolites detected. Upregulated metabolites were defined with fold change ≥2 (red) while downregulated metabolites were with fold change ≤0.5 (green). In addition, a threshold of VIP>1 was applied to distinguish DAMs from the unchanged ones. **(B)** Metabolites with the highest VIP score. **(C)** Metabolites with the highest log2-transformed fold change. **(D)** The top 20 pathways with the lowest *p*-values. Rich factor is the ratio of the number of DAMs to all metabolites that were annotated to a pathway. The color of the dots represents level of enrichment, varying from red (*p* = 0) to purple (*p* = 1). The size of the dots indicates the number of DAMs annotated to a pathway.

DAMs between the Luchuan and Duroc breeds were filtered according to their fold change and VIP score, as mentioned in the Methods section. A total of 148 significantly DAMs were identified between the two porcine breeds. 77 metabolites were downregulated while 71 were upregulated in Luchuan compared to Duroc (Figure 2A). Metabolites with top 20 highest VIP score and log2-transformed fold change were displayed (Figures 2B, C). Some of the most significantly altered metabolites were listed below and were sorted by their Log2 fold change (Table 1).

**TABLE 1:** Statistics of top 20 most significantly upregulated and downregulated metabolites in Luchuan and Duroc skeletal muscle (Sorted by log2-transformed fold change, VIP>1).

Metabolites	Log2 fold change	VIP	*p*-Value	Type
Glycerol 3-phosphate	4.251	1.692	0.052	Up
9(S)-HODE	3.913	1.656	0.068	Up
Cis-9,10-epoxystearic acid	3.240	1.964	0.003	Up
Carnitine ph-C1	3.004	1.931	0.010	Up
12,13-DiHOME	2.766	2.077	0.003	Up
D-Fructose-1,6-Biphosphate-Trisodium Salt	2.531	1.570	0.040	Up
Arachidic Acid (C20:0)	2.355	1.737	0.044	Up
13-HpODE	2.326	1.692	0.049	Up
Mannose 1-phosphate	2.207	1.281	0.021	Up
Valyl-leucine	2.050	1.469	0.007	Up
Deoxyguanosine	−4.920	1.534	0.018	Down
N-Methyl-L-Glutamate	−3.536	1.978	0.022	Down
2′-Deoxyinosine	−3.008	1.696	0.005	Down
2-Aminoadipic Acid	−2.954	1.734	0.024	Down
N-Acetyl-L-Leucine	−2.939	1.430	0.100	Down
Thymine	−2.725	1.325	0.006	Down
Gamma-Mercholic Acid	−2.604	1.447	0.001	Down
2-Pyrrolidinone	−2.438	1.495	0.012	Down
L-Cystathionine	−2.415	1.395	0.016	Down
Uric acid	−2.324	1.178	0.013	Down

The significant DAMs were annotated and mapped to the KEGG database. We summarized the number of metabolites that were mapped to each of the pathways. The ABC transporters (ATP-binding cassette transporters), accountable for 22.5% of all the significant metabolites, are a protein superfamily involved in a large variety of metabolic processes, for instance, the translocation of lipids and sugars. Some pathways related to growth such as protein digestion and absorption, thyroid hormone signaling and thyroid synthesis, are slightly augmented.

Subsequently, KEGG pathway enrichment analysis was performed to identify the pathways that contributed to the variance of metabolic profiles between the two porcine breeds. No pathway was enriched at a statistically significant level (*p* < 0.05) in our metabolomic analysis. We demonstrated the top five pathways with the lowest non-significant *p*-values (*p* < 0.1), which are fructose and mannose metabolism, biotin metabolism, biosynthesis of unsaturated fatty acids, autoimmune thyroid disease, and alpha-linolenic acid metabolism (Figure 2D).

### Comparison of metabolic profiles between Luchuan and Duroc

Among all the DAMs we identified, 9 lipids, 18 glycerophospholipids and 10 fatty acids from Luchuan were substantially higher than in Duroc. Carnitines (especially carnitine C4:0 and carnitine C5:1), punicic acid, 13 (R)-HODE and LysoPCs (Lysophosphatidylcholines, especially LysoPC 18:2) showed the highest concentration under the Fatty Acids (FA), Oxidized Lipids and Glycerophospholipids (GP) categories. 22 amino acids and their derivatives were significantly lower in Luchuan compared to Duroc. L-aspartic acid and L-glutamic acid together made up the majority of differentially accumulated amino acids; N, N-Dimethylglycine, N-Methylalanine and aminoisobutyric acid were the most enriched amino acid derivatives.

We found that 14 organic acids and their derivatives were downregulated in Luchuan. The concentration of citric acid, succinic anhydride and L-2-aminobutyric acid exceeded other organic acids or derivatives by far. In Luchuan, 10 sugars were reduced while 8 were increased. D-glucose 6-phosphate and D-mannose 6-phosphate were most abundant. Fructose and mannose metabolism is one of the metabolic pathways with lowest *p*-values in our metabolome KEGG result. Mannose-1-phosphate, the most elevated metabolite under the sugars and sugar phosphates class, is in the center of fructose and mannose metabolism. In addition, 9-octadecenal, an aldehyde associated with meat flavor and aroma (Calkins and Hodgen, 2007), was significantly elevated in Luchuan.

### Transcriptome analysis

**FIGURE 3:**
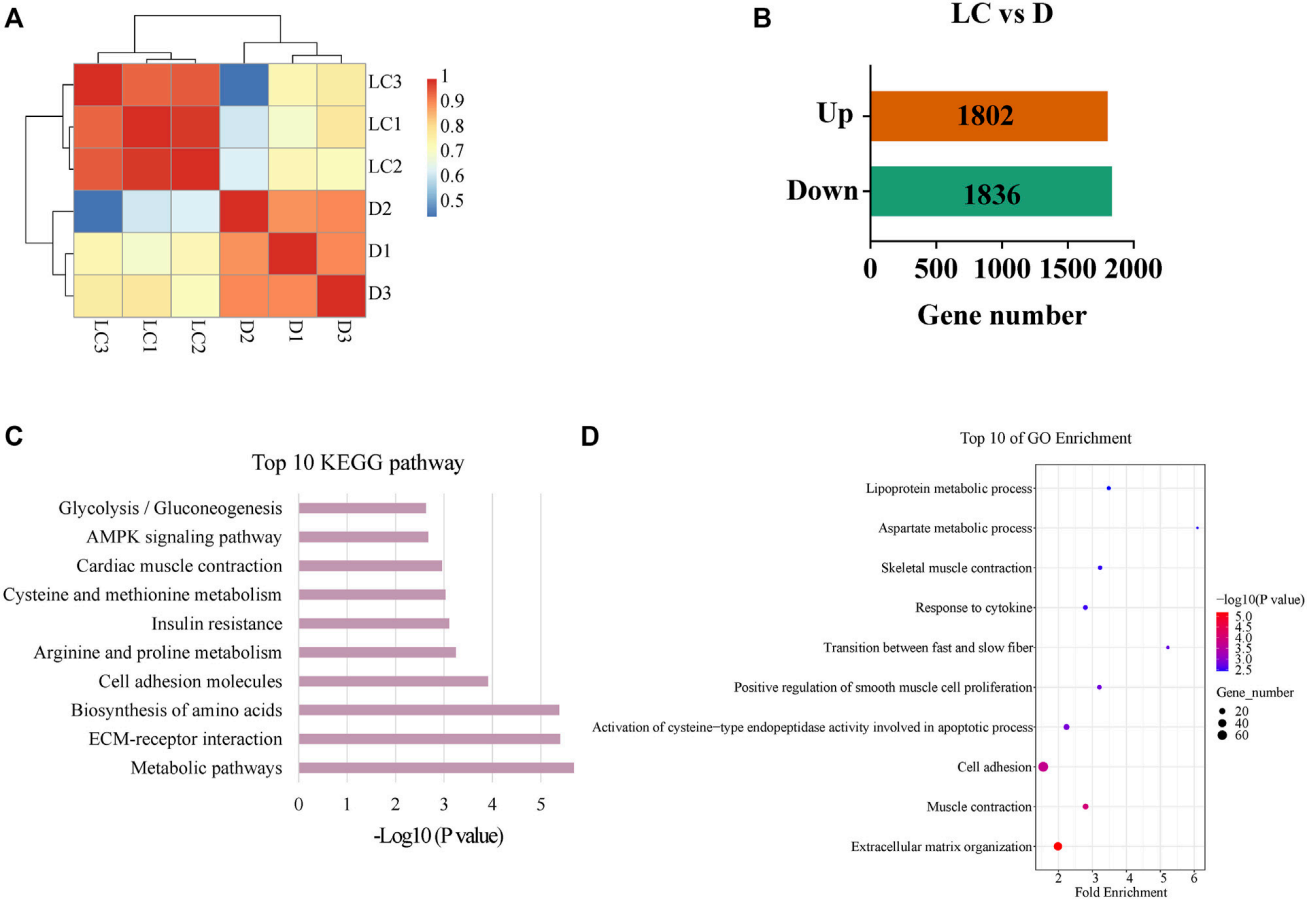
Analysis of DEGs between Luchuan and Duroc. **(A)** Correlation of gene expression of DEGs based on TPM. **(B)** Number of upregulated and downregulated DEGs. **(C)** Top 10 most enriched pathways of the DEGs based on *p*-value. **(D)** Top 10 GO enrichment terms based on *p*-value; the size of dots indicates number of genes related to the term.

High throughput transcriptome sequencing was performed using the longissimus dorsi muscle of Luchuan and Duroc pigs. We first calculated the correlation of gene expression among individuals (Figure 3A), and the results showed higher correlation of gene expression between individuals from the same breed. Next, a differential analysis was done to identify DEGs between Duroc and Luchuan. We identified a total of 22,909 genes of which 1802 were upregulated and 1836 were downregulated in Luchuan compared to Duroc (Figure 3B). We further examined these DEGs using GO and KEGG enrichment analyses, and the result demonstrated similar pathways and annotations as the analysis for the metabolome. The top 10 most significantly enriched KEGG pathways (*p* < 0.05) are shown in Figure 3C. Noticeably, pathways related to glucose metabolism and fatty acid uptake and oxidation such as “Glycolysis/Gluconeogenesis” and “AMPK signaling pathway”, pathways related to amino acid metabolism such as “Arginine and proline metabolism” and “Biosynthesis of amino acids”, are enriched. The GO enrichment revealed that many of the DEGs are related to muscle contraction, muscle development and cell adhesion (Figure 3D).

### Integration analysis of metabolome and transcriptome

**FIGURE 4:**
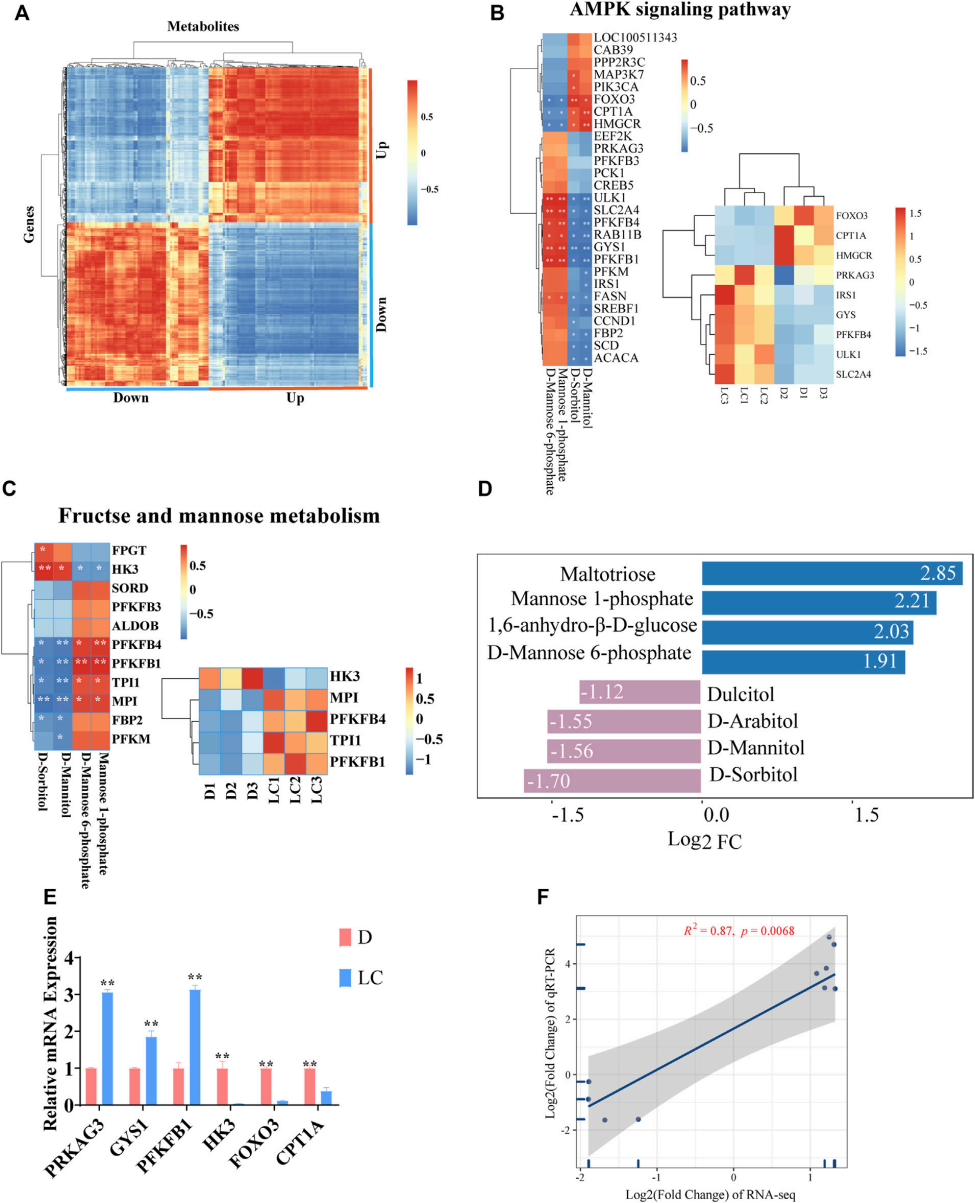
Integrative analysis of metabolome and transcriptome. **(A)** Heatmap of correlation between all upregulated and downregulated DEGs and DAMs. **(B)** Correlation between genes and metabolites, and the expression level of several key genes in AMPK signaling pathway in different individuals. **(C)** Correlation between genes and metabolites, and the expression level of several key genes in fructose and mannose metabolism in different individuals. **(D)**The Log2 Fold Change (FC) of several metabolites involved in these two pathways. **(E)** Relative mRNA expression level (qPCR) of several genes that are essential in these two pathways. **(F)** Linear regression between Log2 FC of gene expression (RNA-seq) and Log2 FC of relative mRNA expression (qPCR).

In order to identify genes that can contribute to changes in metabolites and thus affect phenotypes, we first correlated all DAMs and DEGs (Figure 4A). A strong positive correlation was observed in upregulated DAMs against upregulated DEGs, and *vice versa*. A strong negative correlation was observed in upregulated DAMs against downregulated DEGs, and *vice versa*. We then chose two pathways, AMPK signaling pathway and fructose and mannose metabolism, which were present in both of our metabolomic and transcriptomic analyses as our primary focus. Both pathways play important roles in glucose uptake, glycolysis and energy metabolism. D-Mannitol and D-Sorbitol are the shared metabolites, while *PFKFB1* and *PFKFB4* are the shared significant genes between these two pathways (Figures 4B–D). The expression of genes was examined using qRT-PCR, and the results meet our expectations. *PRKAG3*, *ULK1*, *GYS1*, *TPI1*, *PFKFB1*, *FASN*, *MPI* and *IRS1*, genes significantly upregulated in Luchuan based on our RNA-seq data, also present higher mRNA expression in our qPCR test. Similarly, *HK3*, *HMGCR*, *CPT1A* and *FOXO3* have significantly lower expression levels in both of our RNA-seq and qPCR results (Figure 4E and Supplementary Figure S1). Linear regression was applied to check the connection between Log2 fold change of RNA-seq and Log2 fold change of qRT-PCR, and strong positive correlation (*R* ^2^ = 0.87) was observed (Figure 4F).

## Discussion

We applied UPLC-MS based widely targeted metabolomics to identify and quantify low weight molecules and metabolites from longissimus dorsi muscle of the two pig breeds Luchuan and Duroc. Revealing the chemical composition of skeletal muscle provides clues to the energy metabolism, growth, and meat characteristics in animals. We managed to obtain total ions current (TIC) chromatograms and multimodal maps detected by MRM from our UPLC-MS experiments. A total of 609 metabolites were identified and quantified, 148 of which were significantly altered between our Luchuan and Duroc samples based on their fold change and VIP score extracted from the OPLS-DA model. Metabolites with a VIP score of more than 1 are considered important for the model, and we focused on those with the highest fold change in our differential, pathway enrichment and integrative analyses. Our multivariate and hierarchical cluster analysis results confirmed substantial intergroup differences between Luchuan and Duroc, indicating that the two pig breeds have not only their distinctive features, but also exclusive metabolic profiles.

Fatty acids, oxidized lipids and several glycerophospholipids were significantly elevated in Luchuan compared to Duroc. The oxidized lipid 12,13-dihydroxy-9Z-octadecenoic acid (12,13-DiHOME), substantially higher in Luchuan, has the highest VIP score (2.077) among all DAMs. It was reported that 12,13-DiHOME can promote fatty acid transport into brown adipose tissue (Lynes et al., 2017), and be able to facilitate skeletal muscle fatty acid uptake (Stanford et al., 2018). Several carnitines, which play critical roles in energy production and chemical transportation, were found upregulated. Carnitines are normally concentrated in tissues that utilize fatty acids as dietary fuel since they can transport long-chain fatty acids into the mitochondria (Rebouche et al., 1999). Meanwhile, our KEGG result revealed a few pathways related to fatty acid synthesis and lipid metabolism, including alpha-linolenic acid metabolism, biotin metabolism and biosynthesis of unsaturated fatty acids (*p* < 0.1). PPAR signaling pathway and ABC transporters, closely related to energy metabolism and lipid transportation (Barger and Kelly, 2000; Ide et al., 2003; Kennedy et al., 2005; Ito et al., 2012), were likewise found in our KEGG result (*p* < 0.25).

Various amino acids and their derivatives, such as L-aspartic acid and L-glutamic acid, which are common non-essential amino acids for human, were considerably decreased in Luchuan. N-methyl-L-glutamate, derived from L-glutamic acid, has the second highest VIP score (1.978), and was significantly reduced in Luchuan as well. Glutamic acid is the major excitatory neurotransmitter conducive to neuronal differentiation, migration, and survival in the developing brain (Tapiero et al., 2002). It can be converted to glutamine, which is the substrate and precursor for many metabolic processes such as nucleotide and nucleic acid synthesis. Aspartic acid is the precursor of several amino acids, and it can participate in various biosynthetic pathways. In eukaryotes, the malate-aspartate shuttle is an important biological system for translocating electrons during glycolysis. We observed a significantly lower level of urea in Luchuan, which is one of the end products of purine and pyrimidine metabolism. The nucleic acids and nucleotides altered were mostly downregulated in Luchuan. Deoxyguanosine, the final product of GTP catabolism, showed the highest fold decrease of all metabolites. All this suggests a potentially lower level of amino acid and nucleic acid metabolism in Luchuan. Growing evidence shows that some amino acids are important regulators of key metabolic pathways that are necessary for maintenance, growth, reproduction, and immunity in animals, therefore maximizing efficiency of food utilization, enhancing protein accretion, reducing adiposity, and improving health (Wu, 2009). The amino acids that were downregulated in Luchuan could potentially participate in muscle protein synthesis and degradation, and hereby affecting meat quality by regulating important pathways of fatty acid metabolism and fiber characteristics in the skeletal muscle. Meanwhile, nucleotides are required for a wide variety of biological processes. When cells proliferate, increased nucleotide synthesis is necessary for DNA replication and for RNA production to support protein synthesis at different stages of the cell cycle (Lane and Fan, 2015). Decreased level of nucleic acid metabolism could be coupled with decreased level of muscle protein synthesis, and therefore have a substantial impact on muscle growth.

We identified 3638 DEGs based on our RNA-seq data, which account for 15.9% of all annotated genes in the reference genome. The number of DEGs is substantially larger than observed in some of the other transcriptome studies between various pig breeds (Song et al., 2019; Zhang et al., 2021), but the proportion of upregulated genes and downregulated genes in our research is roughly the same as in these studies. A stronger correlation in gene expression is observed between individuals within the same breed compared to individuals from both breeds, which coincides with our observation in the metabolic profile that the Luchuan and Duroc pigs are very diverse. Several of the top enriched pathways of DEGs are shared between transcriptome and metabolome. Our GO analysis revealed that muscle contraction and transition of muscle fiber type are highly enriched. Glycolysis and fatty acid oxidation are the essential pathways generating energy for basic functions of skeletal muscle (Kerth, 2013). Difference in these critical energy metabolic pathways will likely affect muscle contraction, and potentially lead to changes of growth and meat quality associated phenotypes. Slow-twitch (type I) and fast-twitch (type II) muscle fibers are basic structures of skeletal muscle. Fast-twitch fibers contract faster and are larger in size compared to slow-twitch fibers. Differences in muscle fiber composition can alter overall fiber diameter, fiber density and fiber cross-sectional-area, and therefore significantly impact meat quality (Huo et al., 2021). Furthermore, it has been reported that muscle fiber composition can influence athletic performance in both humans (Zierath and Hawley, 2004) and in animals (Rivero et al., 1993), because Type I muscle fibers display a relatively higher muscular efficiency (Horowitz et al., 1994).

For our combined analysis, we selected two pathways presented in both metabolome and transcriptome: the AMPK signaling pathway and the fructose and mannose metabolism pathway. AMPK is key in maintaining skeletal muscle energy homeostasis, and we observed an elevated level of *PRKAG3* (encodes a regulatory subunit of AMPK) in Luchuan. AMPK activates lipid catabolism by promoting fatty acid oxidation (Dzamko and Steinberg, 2009) and inhibiting the *de novo* biosynthesis of fatty acids and triglycerides (Jeon, 2016). At the same time, AMPK can stimulate glucose uptake and glycolysis by activating *PFKFB* (Marsin et al., 2000; Marsin et al., 2002), while inhibiting glycogen synthesis (Leclerc et al., 2001; Koo et al., 2005). This is confirmed by our RNA-seq and qPCR result that *PFKFB1* and *PFKFB4* are upregulated while *HMGCR* and *FOXO3* are downregulated in Luchuan. However, we observed that the expression of *GYS1* (glycogen synthesis) and *FASN* (fatty acid synthesis) is higher, while that of *CPT1A* (fatty acid oxidation) is lower in Luchuan, suggesting that there could be excessive energy uptake or potential balancing mechanisms in glucose and lipid metabolism in obese types of pigs. Fatty acid synthesis is essential for the IMF content, yet the effect of fatty acid oxidation in obese individuals is still debatable (O'Neill et al., 2013). Fructose and mannose metabolism is closely linked with glycolysis and can provide substrates for sugar nucleotide synthesis. The interconversion of fructose-6-phosphate and mannose-6-phosphate is decisive in maintaining the balance of substrates required for glycolysis and glycosylation reactions. We examined 5 key DEGs and the qRT-PCR results supported our RNA-seq data, showing that *PFKFB1*, *PFKFB4*, *MPI* and *TPI1* are activated while *HK3* is suppressed. Our integration analysis of metabolome and transcriptome revealed a considerably higher level of glycolysis and lower level of fatty acid oxidation in Luchuan, which implies energy utilization can play an important role in determining the unique features in an obese pig breed.

In general, the chemical compounds that vary between the two breeds reflect a critical divergence in metabolism and physiology. In addition, fatty acids and amino acids are notably related to the nutritional value of pork, which indicates that the nutritional properties of these two breeds are highly diverse. Differential expression and pathway enrichment analysis indicated that glucose and lipid metabolism can be instrumental in separating the two selected breeds. Further studies are required to determine how changes in genes and metabolic pathways contribute to the phenotypic variation between the Western lean breeds and Chinese indigenous obese breeds. Comparison of metabolome and transcriptome can promote our understanding to the inner differences of these breeds, which will be beneficial for cultivating and selecting pigs with the desirable features in the future.

## Conclusion

This study revealed substantial metabolic and transcriptomic differences in the skeletal muscle of Luchuan and Duroc pigs, and a considerable number of DAMs and DEGs were identified. The pathway enrichment analysis indicated that glucose and lipid metabolism are responsible for the majority of the differences in metabolome and gene expression between these two breeds. Energy utilization likely determines the distinctive metabolic, physiological and nutritional characteristics in the skeletal muscle of these two breeds. Studying the association of a combination of metabolome, transcriptome and genome with variation in phenotypes of pig breeds will provide insight in the potential molecular mechanisms underlying these complex traits.

## Data Availability

The data presented in the study is deposited in Figshare: https://doi.org/10.6084/m9.figshare.22132949.v7.
