# Supplementary material for: Integrative metabolomic and transcriptomic analysis reveals difference in glucose and lipid metabolism in the longissimus muscle of Luchuan and Duroc pigs
Source: Front Genet. 2023 Apr 6;14:1128033. doi: 10.3389/fgene.2023.1128033 (PMC10118036; doi:10.3389/fgene.2023.1128033)
Supplement: Supplementary file 1 [file DataSheet1.docx]

**Table S1**: qRT-PCR primer sequence.

| Gene name | Primer sequence |
| --- | --- |
| HK3-F | TGGGACCCTCTACAAGCTACA |
| HK3-R | TTGCCAGACCCGTCCTTT |
| TPI1-F | CGCAGATAATGTGAAGGACTG |
| TPI1-R | AAATGATGCGGGTGCTATG |
| PFKFB1-F | ATCTCAAGTGCCCTCTGC |
| PFKFB1-R | GGTTCTCGGGTGATGTCTA |
| PRKAG3-F | ACCTGGCTGCCCAACAAA |
| PRKAG3-R | ACAATCCGGTCAATGACTTCC |
| IRS1-F | GCAACCAGAGTGCCAAAGTGA |
| IRS1-R | ACTGGGTGTCGAGGAGAAGGT |
| GYS1-F | ATGACTCCTCGGACCCTATCT |
| GYS1-R | CTTGACCCTGTCGGCACTA |
| FOXO3-F | ATGGATGCTGACGGGTTGG |
| FOXO3-R | GGCCTGCTTAGCACCAGTGAA |
| CPT1A-F | ACGTGGATTTCCATTCCTTT |
| CPT1A-R | GGGCTGGTTCTGCATTTC |
| HMGCR-F | GCAGATTTGCTCGCCTAC |
| HMGCR-R | TGACCTGGACTGGAAACG |
| ULK1-F | ACAGCAAAGGCATCATCCAC |
| ULK1-R | CGAAGCCGAAGTCAGCAAT |


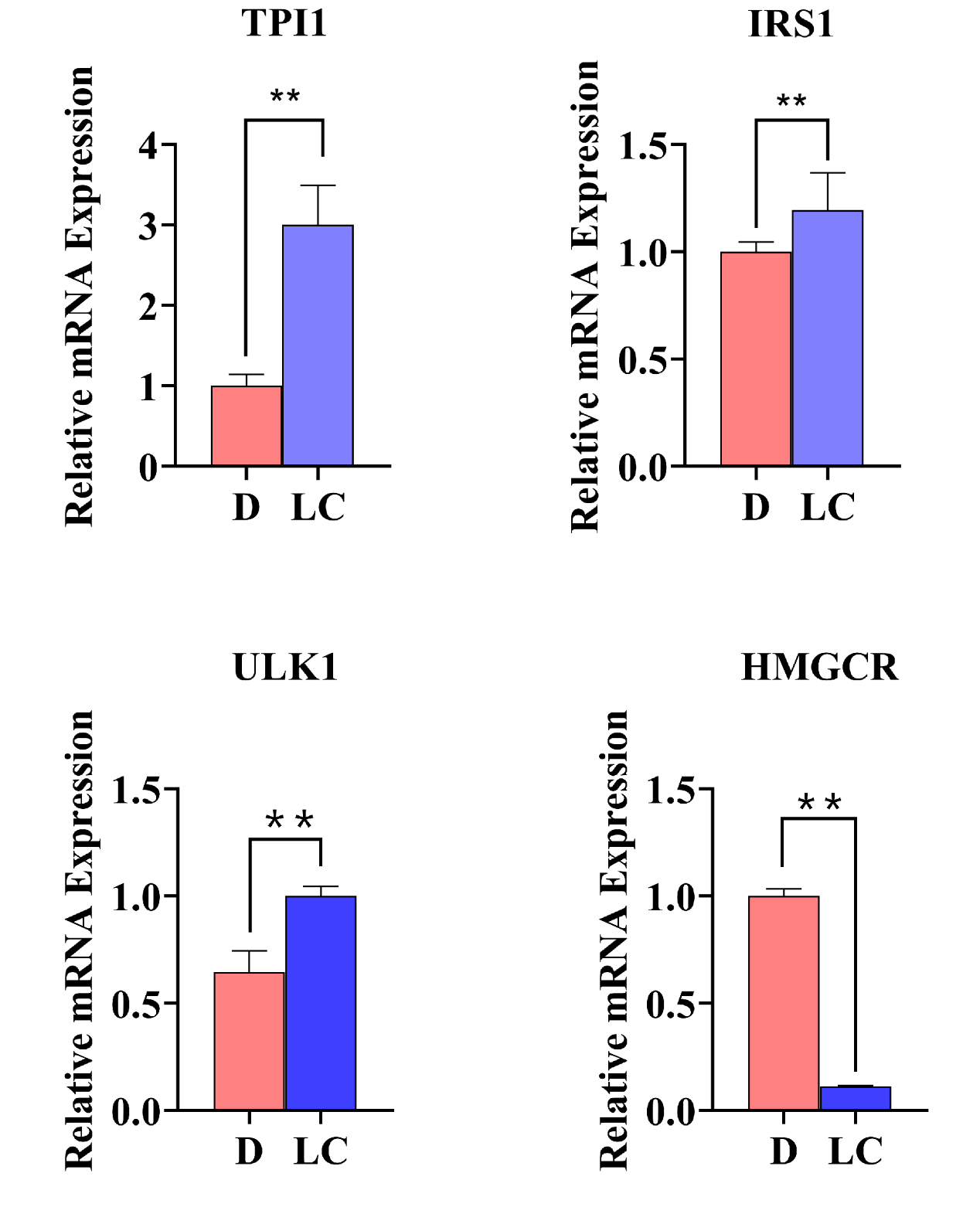


**Figure S1:** Gene expression levels quantified by qRT-PCR. The data are expressed as mean±SD. * *p* < 0.05. ** *p* < 0.01.
